# Shift in a Large River Fish Assemblage: Body-Size and Trophic Structure Dynamics

**DOI:** 10.1371/journal.pone.0124954

**Published:** 2015-04-22

**Authors:** Kyle J. Broadway, Mark Pyron, James R. Gammon, Brent A. Murry

**Affiliations:** 1 Institute for Great Lakes Research, Biology Dept., Central Michigan University, Mount Pleasant, MI 48858, United States of America; 2 Department of Biology, Ball State University, Muncie, IN 47306, United States of America; 3 Department of Biology, DePauw University, Greencastle, IN 46135, United States of America; National University of Mongolia, MONGOLIA

## Abstract

As the intensity and speed of environmental change increase at both local and global scales it is imperative that we gain a better understanding of the ecological implications of community shifts. While there has been substantial progress toward understanding the drivers and subsequent responses of community change (e.g. lake trophic state), the ecological impacts of food web changes are far less understood. We analyzed Wabash River fish assemblage data collected from 1974-2008, to evaluate temporal variation in body-size structure and functional group composition. Two parameters derived from annual community size-spectra were our major response variables: (1) the regression slope is an index of ecological efficiency and predator-prey biomass ratios, and (2) spectral elevation (regression midpoint height) is a proxy for food web capacity. We detected a large assemblage shift, over at least a seven year period, defined by dramatic changes in abundance (measured as catch-per-unit-effort) of the dominant functional feeding groups among two time periods; from an assemblage dominated by planktivore-omnivores to benthic invertivores. There was a concurrent increase in ecological efficiency (slopes increased over time) following the shift associated with an increase in large-bodied low trophic level fish. Food web capacity remained relatively stable with no clear temporal trends. Thus, increased ecological efficiency occurred simultaneous to a compensatory response that shifted biomass among functional feeding groups.

## Introduction

Community shifts are dramatic changes in community composition [1], in response to intense disturbance, or chronic incremental natural or anthropogenic stress [2,3]. Recent research on community shifts has tended to focus on ecosystem monitoring and prediction [3–7]. Fewer studies have examined the effects on ecosystem functioning [8–11], and in particular, few have addressed community body-size distributions and impacts on food web properties before and after major community shifts [12].

Understanding community shift impacts upon food web properties is critical due to increasing anthropogenic disturbances pushing ecosystems beyond their present state [13]. Communities exposed to incremental natural and/or anthropogenic changes such as eutrophication, climate and temperature fluctuations, exploitation, pollution, or non-native species, can result in gradual or sudden shifts from original composition to a contrasting new state [4,11,14]. In some cases the cause of community shifts are quite clear, as the loss of sea urchins from disease led to the collapse of Jamaican coral communities [1,15]. In many cases, however, specific drivers of system change cannot be identified or require complex methods to identify among a suite of acute and chronic stressors [16], or often times data are simply unavailable. Regardless of the factors driving the community changes, shifts clearly result in novel and challenging management scenarios [1,17]. Therefore it is imperative that we develop a better understanding of food web responses. Theory suggests that alternative states may arise as communities respond to perturbations and maintain energetic resilience [11,14,18,19]. These transformations in community composition following impacts may have measurable effects on community body-size distribution, trophic structure, and related food web properties.

Body-size strongly influences population and community dynamics in aquatic ecosystems [20–22]. Patterns of food web body-size structure are a product of predator-prey relationships, gape limitations, and energy dynamics [20,21]. Trophic position often increases with body-size while abundance generally decreases [23–26]. The distribution of abundance or normalized biomass by body-size was long considered an invariant relationship in aquatic ecosystems [24,27]. However, perturbations including over-exploitation, climate change, invasive species, or alteration of nutrient availability have the capacity to alter this relationship [28–30], although some aquatic food webs appear resistant to ecological perturbations [31].

Individual-based community size spectra (CSS) [26,29,32] are log x log regressions that describe the relationship between body size and abundance within a community. CSS are mathematical and graphical representations of traditional pyramids of life [26] and provide two indices of food web function. First, the regression slope represents an index of food web efficiency, an integration of the predator-prey mass ratio and trophic level energy transfer efficiency [26,33]. Second, the regression elevation (centered y-intercept) is a proxy for food web capacity or the abundance/biomass supported within the system [34–36]. Previous CSS studies have analogously termed the CSS elevation as y-intercept [24,37], spectral height [29,38] or midpoint height [35]. CSS are used as indicators of ecosystem well-being as previous studies found responses in spectral properties to natural and anthropogenic stressors [29,31,39,40]. For example, CSS slopes closer to one are suggested for oligotrophic aquatic systems [41] and for communities with organisms of differing body-size competing for similar resources [42], such as large-bodied animals that feed on basal resources. Alternatively, communities dominated by small organisms with atypically low predator-prey ratios due to natural (slowed/stunted growth from intense competition and/or inadequate resource availability) or anthropogenic (overexploitation, selective harvest of large individuals) stressors generally exhibit higher slopes [35,40]. We predicted that changes in assemblage composition, and in particular functional group dominance, are likely to influence food web attributes that we assessed with size spectra metrics.

The Wabash River is a large (watershed of 85,000 km^2^) Midwestern U.S. river that contains a warm-water fishery historically well-known for its abundant fish [43,44]. However, the ecological integrity of the Wabash River has been threatened by increased agriculture, reservoir release, manufacturing activities, urban impacts, and invasive Asian carp in the watershed during the 20^th^ and 21^st^ centuries [43–45]. A directional or predictable trajectory was detected from an analysis of a 25-year (1974–1998) record of fish assemblage structure [46]. Improvements in the form of increased species richness and higher abundances of sensitive taxa were detected in recent fish collections indicating some recovery from decades of ecological degradation [46,47]. Details of the current Wabash River fish assemblage, physical habitats, and hydrologic variability are well-documented [44,48,49]. However, the stability of food web attributes of a large river fish assemblage before and after a major community shift has not been previously examined.

Our analysis consisted of CSS complimented with trophic compositional analyses (functional feeding groups) to assess how community body-size and trophic structure of the Wabash River fish assemblage changed during a 34-year period (1974–2008) which included a clear compositional change in trophic group dominance. The objectives of the investigation were to (1) document a community shift in a large river fish assemblage, and (2) assay the temporal stability of CSS derived indices of food web properties (ecological efficiency and food web capacity), and (3) quantify the relationship between functional feeding group structure and indices of food web properties (CSS metrics). In addition we asked, given the observed changes in assemblage structure of the Wabash River fishes, how do the metrics of food web properties (capacity and efficiency) change?

## Methods

The annual fish surveys conducted by Gammon [43] provided the data for 1974–1998, and additional data were obtained for 2001–8 [46]. Fishes were collected in annual surveys during Jun.–Oct. from 1974–1998 via boat electrofishing with a Smith-Root Type IV GPP (Smith Root Inc., Vancouver, WA, U.S.A.) and in 2001–2008 with a Smith-Root 5.0 GPP with direct current voltage. Boat electrofishing is an effective sampling method due to the Wabash River discharge variation, abundant submerged debris, and steep river banks. The bathymetric heterogeneity prevented other collection methods [50]. The mean gradient (0.12 m km^-1^) and habitat (e.g., poor riffle-pool development, run habitat with gravel, cobble, sand, and silt substrata) were similar for collection locations during all time periods [44,51,52]. Transects where fish were collected were primarily in the middle river reaches from river km 300–530, and were 500-m in length along the outer bend shoreline [44,46,52,53]. The 500-m transect distance was based on an asymptote in species richness [43]. Fishes were identified to species, measured (total length and weight), and released. Fishes were assigned to functional feeding groups (FFG) based on Frimpong and Angermeier’s [54] fish trait database (Table 1). Annual percent composition and catch-per-unit-effort (CPUE; count/electrofishing transect) were calculated for each FFG.

**Table 1:** Species assignments to functional feeding groups and percent abundance before and after assemblage shift in 1992.

FFG	Common Name	Genus species	Pre-shift	Post-shift
Benthic Invertivore	Black Redhorse	*Moxostoma duquesnei*	0.05	0.49
	Blue Sucker	*Cycleptus elongatus*	1.08	2.33
	Freshwater Drum	*Aplodinotus grunniens*	2.31	27.37
	Golden Redhorse	*Moxostoma erythrurum*	1.38	1.16
	Northern Hogsucker	*Hypentelium nigricans*	0.22	0.54
	Quillback Carpsucker	*Carpiodes cyprinus*	0.30	0.32
	River Carpsucker	*Carpiodes carpio*	3.30	13.97
	River Redhorse	*Moxostoma carinatum*	0.04	0.35
	Shorthead Redhorse	*Moxostoma macrolepidotum*	2.1	4.41
	Shovelnose Sturgeon	*Scaphirhynchus platorhynchus*	0.53	0.71
	Silver Redhorse	*Moxostoma anisurum*	1.16	3.03
	Smallmouth Buffalo	*Ictiobus bubalus*	0.0	2.94
	Spotted Sucker	*Minytrema melanops*	0.004	0.0
	White Sucker	*Catostomus commersonii*	0.008	0.0
General Invertivore	Bigmouth Buffalo	*Ictiobus cyprinellus*	0.48	0.98
	Black Buffalo	*Ictiobus niger*	0.03	1.17
	Bluegill	*Lepomis macrochirus*	0.008	0.02
	Goldeye	*Hiodon alosoides*	1.88	0.09
	Green Sunfish	*Lepomis cyanellus*	0.0	0.02
	Longear Sunfish	*Lepomis megalotis*	0.0	0.07
	Mooneye	*Hiodon tergisus*	0.72	0.12
	Redear Sunfish	*Lepomis microlophus*	0.01	0.0
Herbivore-detritivore	Grass Carp	*Ctenopharyngodon idella*	0.0	0.28
	Highfin Carpsucker	*Carpiodes velifer*	0.22	0.64
Omnivore	Common Carp	*Cyprinus carpio*	14.13	10.83
	Channel Catfish	*Ictalurus punctatus*	3.93	5.29
	Flathead Catfish	*Pylodictis olivaris*	10.49	7.6
	Goldfish	*Carassius auratus*	0.004	0.0
	Goldfish/Carp Hybrid	*Carassius/Cyprinus*	0.004	0.0
Parasite	Chestnut Lamprey	*Ichthyomyzon castaneus*	0.0	0.03
	Silver Lamprey	*Ichthyomyzon unicuspis*	0.05	0.01
Piscivore	American Eel	*Anguilla rostrata*	0.17	0.0
	Black Crappie	*Pomoxis nigromaculatus*	0.04	0.14
	Blue Catfish	*Ictalurus furcatus*	0.01	0.1
	Bowfin	*Amia calva*	0.31	0.03
	Grass Pickerel	*Esox americanus vermiculatus*	0.008	0.0
	Largemouth Bass	*Micropterus salmoides*	0.20	0.22
	Longnose Gar	*Lepisosteus osseus*	5.40	1.78
	Sauger	*Sander canadensis*	0.52	0.83
	Shortnose Gar	*Lepisosteus platostomus*	5.44	1.45
	Skipjack Herring	*Alosa chrysochloris*	1.04	0.56
	Smallmouth Bass	*Micropterus dolomieu*	0.63	1.18
	Spotted Bass	*Micropterus punctulatus*	0.41	0.54
	Spotted Gar	*Lepisosteus oculatus*	0.02	0.06
	Walleye	*Sander vitreus*	0.05	0.13
	White Bass	*Morone chrysops*	1.95	1.02
	White Crappie	*Pomoxis annularis*	0.27	0.2
	Yellow Bass	*Morone mississippiensis*	0.01	0.0
Planktivore	Bighead Carp	*Hypophthalmichthys nobilis*	0.0	0.32
	Gizzard Shad	*Dorosoma cepedianum*	38.76	5.75
	American Paddlefish	*Polyodon spathula*	0.03	0.05
	Silver Carp	*Hypophthalmichthys molitrix*	0.0	0.87

We used principal component analysis (PCA) (SAS, Proc Princomp) and a simple cumulative sums of deviation from the mean test (CUSUM) of the annual CPUE abundances of FFGs (benthic invertivores, planktivores, omnivores, and piscivores) to identify shifts in community FFG dominance [5]. A shift in FFG distributions was illustrated by changes in the direction (positive or negative) of scores of the dominant principal components through time. Two distinct time periods were identified from the PCA and differences of the major principal components were tested using a random intervention analysis with one million iterations [55]. Functional feeding group composition and CSS metrics (indices for food web characteristics) among the time periods were also evaluated with the random intervention approach. Linear and non-linear regressions were used to evaluate temporal relationships of FFG percent composition. The pseudo-R^2^ was calculated as a relative comparison to the linear models. Similarly, the cumulative deviation from the mean for each functional group increases (or decreases) steadily until a switch point is crossed in which the directional temporal trend changes direction [56]. In our context a humped distribution is indicative of a sustained shift in FFG CPUE and the top of the peak (or low point in the valley) designates the year of the observed shift.

We used a community size-spectra (CSS: log_2_ CPUE x log_2_ body-size regression [29]) approach to estimate the functional food web properties of ecological efficiency and food web capacity. Linear associations of CPUE and body-size resulted from log_2_ transformation [57]. Fishes were assigned to size classes of log_2_ total length. Individuals with total length less than 182-mm are not effectively sampled by boat electrofisher and were not included in the analyses (similar gear efficiency thresholds as others [29,31,35,38]). We calculated the mean log_2_ total length and CPUE for each size class. CSS regressions were created for each year using mean log_2_ total length vs. log_2_ CPUE. Annual CSS were centered on the median size class, 8.5, (mean log_2_ total length—8.5) to remove the correlation between slope and intercept [35,38,58]. Two fundamental food web properties were assessed from annual CSS: (1) the regression slope as an index of ecological efficiency, and (2) the spectral elevation (centered y-intercept) served as a proxy for food web capacity. We examined regressions of dominant FFG percent abundance to examine temporal patterns.

The relationships between annual CSS slope and FFG assemblage composition were modeled with least squares linear regressions and when appropriate nonlinear regression (logistic model using SAS Proc NLIN Newton Method). The nonlinear logistic model goodness-of-fit measure was determined mathematically with the formula pseudo-R^2^ = −(SS error / SS total [corrected]). The pseudo-R^2^ was calculated for relative comparison to the linear regression models. All statistical tests were conducted in SAS 9.8, SAS Studio, and/or R statistical software 3.1.0 with alpha = 0.05.

## Results

**Fig 1:**
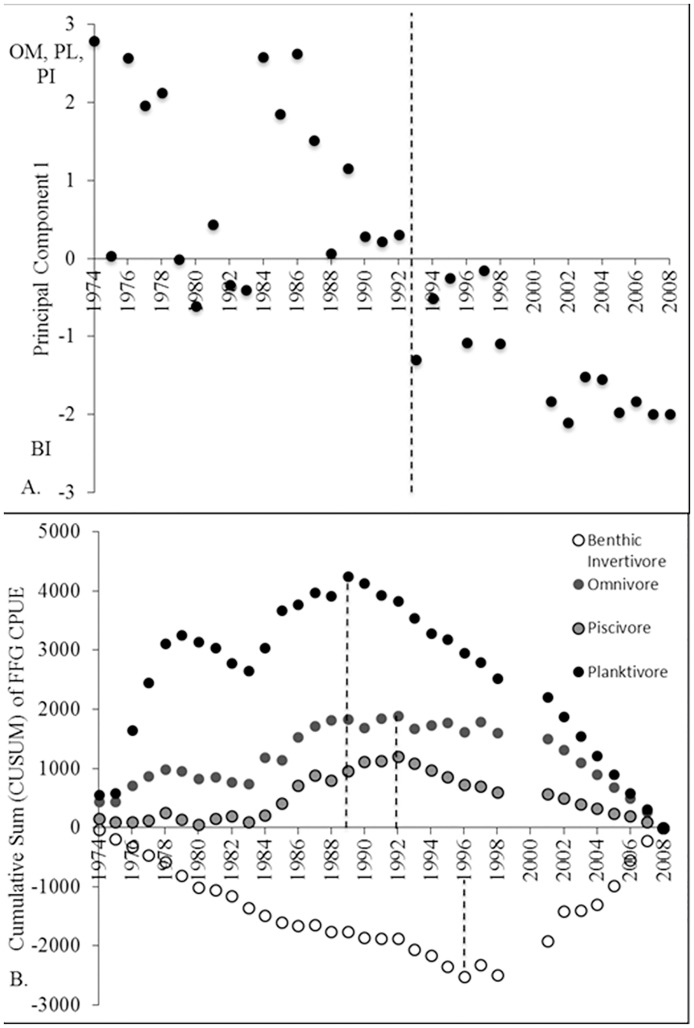
(A) Principal component scores plotted with time to define community shifts. The first principal component described 59.6% of the total variation in annual FFG catch-per-unit-effort. The first principal component contrasts omnivores (OM, factor loading 0.57) and planktivores (PL, 0.56), and piscivores (PI, 0.49) with benthic invertivores (BI, −0.34). The community shift is indicated by the vertical dotted line at 1992–3. (B) The cumulative deviations from the mean for each FFG. Dotted lines illustrate sequential shifts in planktivore relative abundance in 1989, omnivores and piscivores in 1992, and benthic invertivores in 1996.

**Fig 2:**
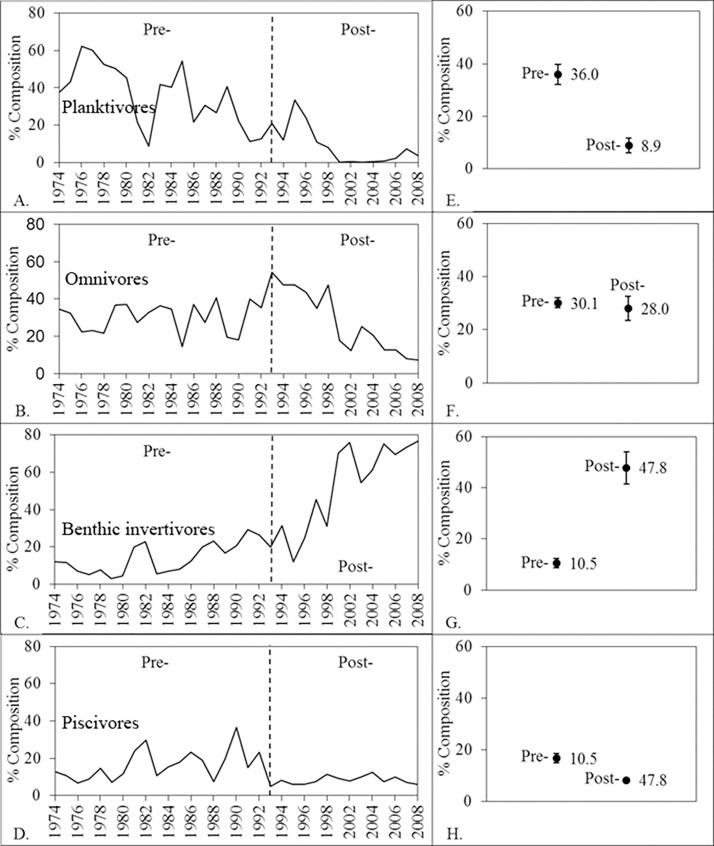
Mean percent composition of planktivores (A,E), omnivores (B,F), benthic invertivores (C,G), and (D,H) piscivores during time periods (1) 1974–1992, and (2) 1993–2008. Variation is indicated by standard error bars.

The dominant fish assemblage FFGs underwent dramatic changes during the 1974–2008 period (Fig 1A; [5,7,9,14]). The first principal component described 59.6% of the total temporal variation in FFG and differed significantly among time periods (random intervention analysis, *P* = < 0.001). The pre-shift (1974–1992) fish assemblage was dominated by planktivores (36.0%), omnivores (30.1%), and piscivores (16.6%). Benthic invertivores represented only 10.5% of abundance in the earlier time period. However, in the post-shift period (1993–2008) benthic invertivores dominated the fish assemblage (47.8%, Table 2, Fig 2), and the composition of the other three functional groups decreased, particularly planktivores (post-regime shift planktivores 8.9%, piscivores 8.1%, omnivores 28.0%, Table 2, Fig 2).

**Table 2:** Mean percent community composition and relative catch-per-unit-effort by functional feeding group, during time periods (1) 1974–1992 and (2) 1993–2008.

Functional Feeding Group	% Composition		CPUE	
	1974–1992	1993–2008	1974–1992	1993–2008
Benthic Invertivore	10.5	47.8	5.9	21.0
Standard Error	0.9	1.5	4.8	7.4
Omnivore	30.1	28.0	18.2	7.0
Standard Error	3.0	1.9	0.9	4.6
Piscivore	16.6	8.1	9.7	2.9
Standard Error	1.3	1.9	0.5	0.6
Planktivore	36.0	8.9	26.9	1.7
Standard Error	7.0	3.8	0.5	2.8

Benthic invertivore CPUE abundance increased by a magnitude of 1.8 from pre- to post-shift periods (random intervention analysis, *P* = 0.0014). Omnivore CPUE abundance was significantly higher in the pre-shift than the post-shift period (random intervention analysis, *P* = 0.0002). Planktivore CPUE abundance during the post-shift period was significantly less than during the pre-shift period (random intervention analysis, *P* < 0.0001). Similarly, piscivore CPUE abundance decreased from the pre-shift to post-shift periods (random intervention analysis, *P* < 0.0001; Table 2). CUSUM analysis illustrated the sequential chain of events that resulted in a complete community shift of the Wabash fish assemblage (Fig 1B). The shift appears to have taken several years, from 1989–1996, to complete and may be related to the general expected life spans or population turnover rate typical of each FFG. The community shift began with a decrease in planktivore abundance in 1989, followed by similar decreasing trends in 1992 for omnivore and piscivore abundances. Finally, the generally larger-bodied and longer-lived benthic invertivores increased in abundance substantially beginning in 1996.

**Fig 3:**
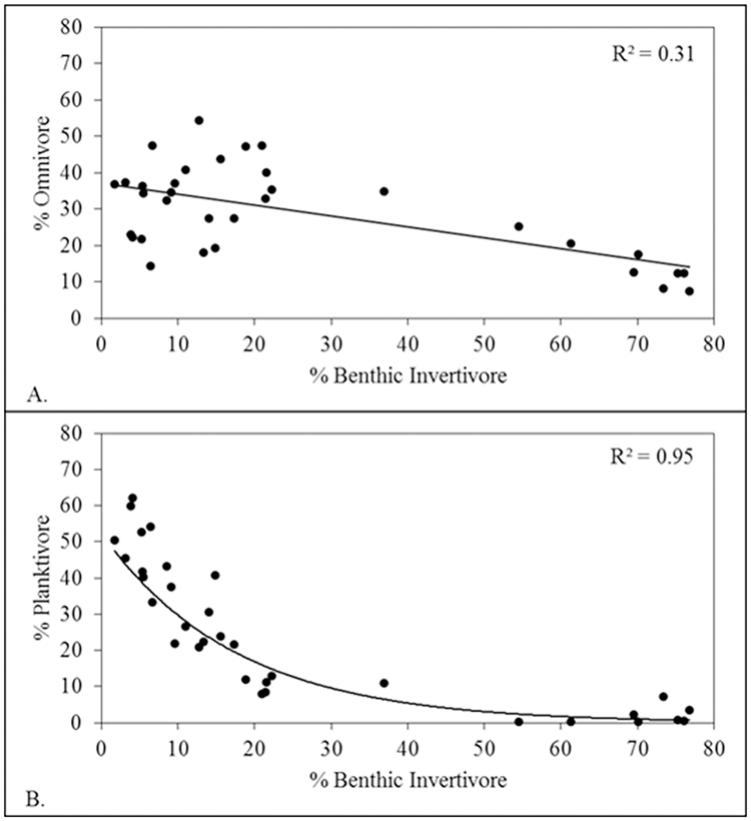
Percent abundances for (A) benthic invertivores and omnivores; and (B) benthic invertivores and. planktivores.

There were strong negative temporal relationships among FFG percent abundances. The percent composition of omnivores decreased linearly with increasing percent composition of benthic invertivores (*r* ^2^ = 0.31, *F*_1, 32_ = 13.63, *P* = 0.0009; Fig 3A) and planktivore percent composition also decreased (nonlinearly) with increasing benthic invertivore composition (pseudo-*r* ^2^ = 0.95, *F*_2, 33_ = 311.63, *P* < 0.0001; Fig 3B). Both omnivores and planktivores had a threshold response when benthic invertivore percent composition reached 25–30% (Fig 3A and 3B).

**Fig 4:**
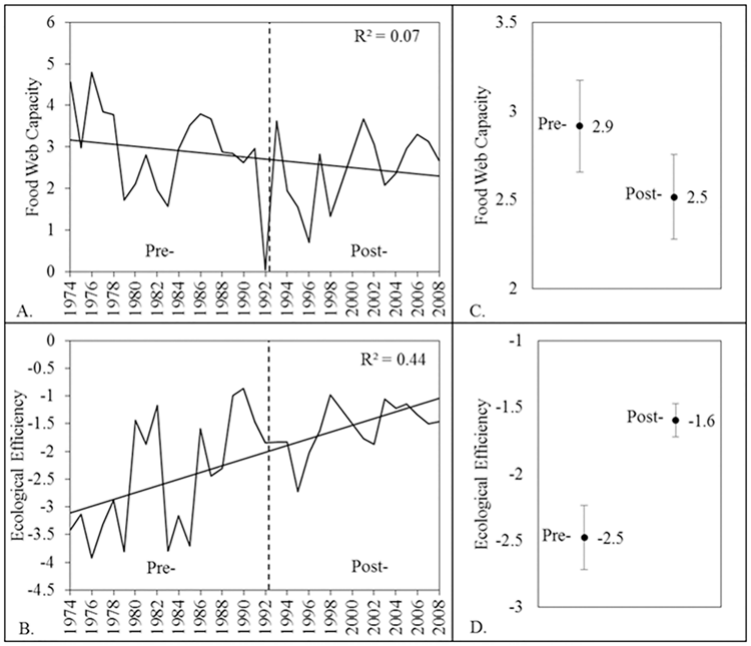
Annual trends of functional food web indices from 1974–2008. (A) food web capacity as a proxy for the centered spectral elevation of annual CSS, (B) ecological efficiency indexed by the regression slope of annual CSS, (C) mean food web capacity during early and late time periods including standard error, and (D) mean ecological efficiency during early and late time periods including standard error.

The food web capacity inferred from annual CSS elevations did not vary significantly among early and late periods (random intervention analysis, *P* = 0.28; Fig 4A) indicating that the ‘size’ of the food web remained similar from 1974–2008. In contrast, the ecological efficiency indexed by the CSS slope increased from the early to late time periods. Annual slopes became less negative (i.e., flatter) and ranged from -3.4 when planktivores dominated the assemblage in 1974, to -1.4 in 2008 when benthic invertivores were dominant (mean pre-shift slope = -2.5 (stdev = 1.1) and mean post-shift slope = -1.6 (stdev = 0.5); Fig 4D). The size spectra slope (index of ecological efficiency) was significantly lower during the early time period than the late time period (*P* = 0.007; Fig 4B–4D).

**Fig 5:**
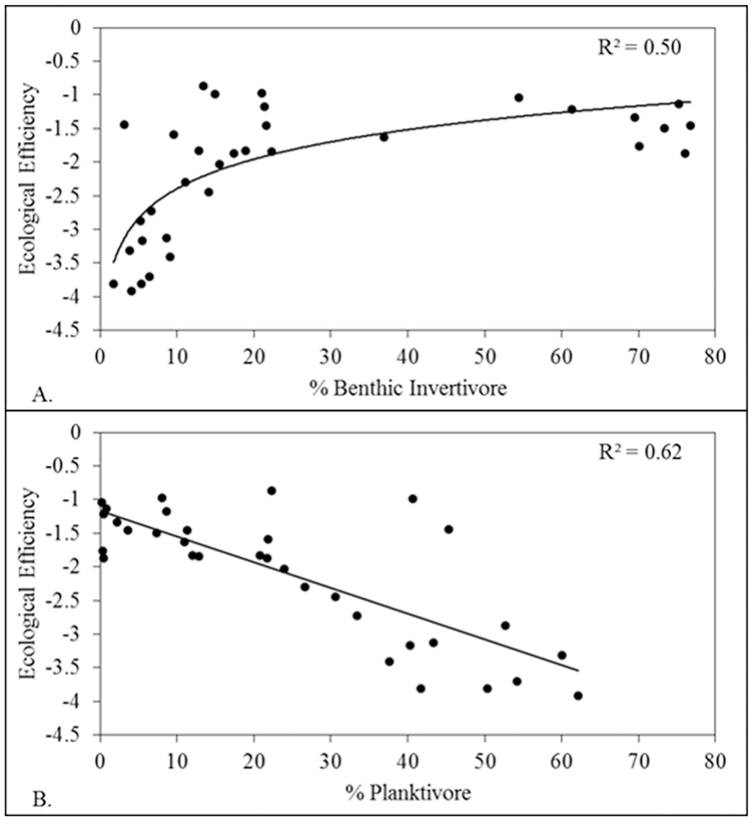
Temporal relationships of ecological efficiency with percent relative abundance for two functional feeding groups: (A) benthic invertivores, (B) planktivores.

Two significant relationships resulted for FFGs and CSS food web properties. First, ecological efficiency increased nonlinearly with increasing benthic invertivore percent relative abundance (pseudo-*r* ^2^ = 0.50, *F*_1, 32_ = 33.26, *P* < 0.0001, Fig 5A), and second, ecological efficiency decreased linearly with increasing planktivore percent relative abundance (*r* ^2^ = 0.62, *F*_1, 32_ = 41.04, *P* < 0.0001; Fig 5B). Ecological efficiency was not, however, significantly related to the percent relative abundance of omnivores or piscivores (*P* > 0.05 in both cases). A clear threshold response of ecological efficiency to benthic invertivore percent relative abundance occurred at relative abundances of approximately 25–30% (Fig 5). Annual food web capacities were not a product of FFG composition (*P* > 0.05 in all cases).

## Discussion

Community size-spectra derived from simple fishery survey data were a useful tool to identify changes in food web structure and function associated with a large shift in FFG dominance. In the Wabash River we observed a change in FFG composition from a planktivore-omnivore-piscivore dominated assemblage to an assemblage grossly dominated by benthic invertivores. Concomitant with the FFG changes were clear variations in food web functionality. The increase in benthic invertivore relative abundance from early to late time periods coincided with a 44% increase in the CSS index of ecological efficiency, and the food web capacity did not result in significant temporal trends.

The distinct changes in community structure we observed suggest a major ecosystem transformation. Regime shifts have been identified in multiple ecosystems [9,11,14]. However, this is the first identification of a large community shift for a large river ecosystem with little flow regulation from mainstem reservoirs [59]. In this situation, the term “regime shift” may not be appropriate in characterizing the shift, as the process occurred during several years rather than abruptly. The change point may be difficult to identify due to high variation in the fish assemblage through-out the collection period. However, two distinct temporal periods were obvious, suggesting a major community shift.

Interestingly, the shift appears to have occurred over at least a seven year period (1989–1996) and followed a clear sequential pattern of shifts in individual FFGs, that may be tied to their life histories and in particular population turnover rate. Planktivores were numerically the dominant FFG prior to the shift, beginning in 1989. This FFG is composed of gizzard shad primarily, and has the shortest population turnover time. Following the planktivores decline we observed subsequent declines in omnivores and piscivores, beginning in 1992. Finally, benthic invertivores, which were historically a relatively minor component of the fish assemblage, expanded greatly beginning in 1996. Benthic invertivores tend to be larger-bodied and longer-lived species, resulting in slower population turnover time. While population turnover time could partially explain some of this observed sequential change, community level compensation is an alternate hypothesis (31). Food web capacity remained relatively stable with time indicating that the total abundance of fish in the river was relatively constant (but its distribution among FFGs varied); thus under a community compensation hypothesis individuals (or biomass) lost from declining FFGs (i.e., planktivores, omnivores, and piscivores) were replaced by benthic invertivores abundance (biomass).

Although we identified temporal structural changes (i.e., shift in FFG dominance), we can only speculate about mechanisms causing an observed Wabash River fish assemblage community shift. Generally, underlying mechanisms promoting these shifts are notoriously difficult to identify, even for systems with detailed long-term monitoring [9]. The difficulty lies with the lack of warning period before the shift with natural systems that have unpredictable dynamics [60]. Gradual human alterations reduce resilience and result in systems that are vulnerable to community shifts [6,9,11,61]. Human alterations of the Wabash River ecosystem include multiple hydrologic impacts: dams on tributaries, agricultural tile drainage to increase flow and removal of water from row crops soil subsurface, urbanization throughout the watershed [48], historical industrial pollution [43], introduction of invasive Asian carp [62], ubiquitous treated and untreated wastewater discharges and combined sewage overflows throughout the watershed [63]. Thus, highly probable anthropogenic stressors capable of impacting the resilience of the Wabash River ecosystem include: (1) varied and high influx of nutrient loads, (2) invasive aquatic species, (3) altered hydrologic regime, or (4) a combination of these and perhaps additional ecosystem stressors (e.g., climate change).

Our analyses of annual CSS elevations (centered y-intercepts) demonstrated that the Wabash fish assemblage food web capacity, while temporally variable, was not measurably impacted by the community shift. The total composition of fishes supported by the food web was clearly limited and similar among time periods. The redistribution of abundances among FFGs, recognizing the limits to food web ‘size’, indicates the occurrence of an assemblage level compensatory response. As the percent relative composition of planktivore and omnivore FFGs declined, a compensatory response was increasing composition of benthic invertivores. The assemblage compensatory response was identifiable from the threshold reaction, when the assemblage approached 25–30% benthic invertivore composition, triggering rapid increase of benthic invertivores and rapid decrease of planktivores and omnivores (Fig 3A and 3B). The assemblage compensatory shift rearranged the trophic structure from a predominantly small-bodied (gizzard shad) assemblage to one dominated by fishes that feed at lower trophic positions but have large asymptotic body size (e.g., freshwater drum, shorthead redhorse, and river carpsucker). This coincided with increasing ecological efficiency in the late time period (Fig 4B).

Previous analyses of a segment of the 1974–98 dataset using abundance information demonstrated a gradual and directional change in fish assemblage trajectory [46], strong temporal correlations using a trait-based or taxonomy-based approach [64], and improvements in biological integrity [65]. Our analyses using body size and FFG information and an increased duration dataset provided additional details that allowed detection of trophic changes. We observed a trend starting in 1984–90 (Fig 5), with increased relative abundances of omnivores and piscivores, planktivore relative abundance was stable, and benthic invertivore relative abundance increased. During this time, JRG observed noticeable increases in abundances of intolerant fish species, simultaneous with increased dissolved oxygen in the water column. This likely was a direct response to enforcement of the Clean Water Act of 1972, with increased effectiveness of treating domestic and industrial waste effluent to the river. River discharge was low, contributing to excellent reproduction for many fishes. The Department of Agriculture’s payment in kind (PIK) program of 1983 likely contributed to improved ecosystem quality [66]. The program paid farmers not to plant grain, with a goal to decrease excess crop stores held by the federal government, resulting in decreased inputs of nutrients and sediments to the river.

The role of species identity in food web and ecosystem functioning is a central question in ecology [67,68]. Though our study was not experimental, we provide observational support of the hypothesis that species functional roles have both linear and non-linear effects on food web functioning. We observed a linear decrease in food web efficiency with increasing composition of planktivorous species (gizzard shad) and a non-linear (logistic) increase in food web efficiency with increasing composition of benthic invertivore species.

Planktivorous fishes, excluding exceptions like the adult invasive Asian carp, are typically small- to mid-sized species that fill middle trophic positions and generally are a prey base for larger piscivorous fishes [69]. Because they consume basal resources and then are subsequently prey for larger fish the planktivores are effectively energy conduits. Each trophic link incurs energy loss and reduces available energy to support higher levels [20]. In contrast, in the Wabash River and many moderate to large temperate rivers, the majority of benthic invertivores are large-bodied low trophic position (LBLTP) fish. LBLTP fish consume basal resources (benthic invertebrates and detritus) and are generally long-lived as they grow rapidly to large asymptotic size, which greatly reduces their susceptibility to predation. LBLTP fish are hypothesized to sequester energy, potentially limiting resource availability to other components of the food web [70]. Basic energetics theory suggests that communities that are dominated by LBLTP species have fewer trophic links and thereby should have higher food web efficiency and the capacity to support higher abundances of large-bodied individuals, compared to communities dominated by piscivorous fish [71]. In the Wabash River ecosystem, food web efficiency resulted in a threshold response to LBLTP (benthic invertivore) composition. Food web efficiency increased with increasing LBLTP composition until the LBLTP fish exceeded roughly 25–30% of all fish after which efficiency stabilized (Fig 5A). During the recent time period, the composition of LBLTP fishes exceeded 75% of all fishes, but there was little increase in food web efficiency between 30% and 75% dominance of LBLTP fish. There appears to be an ecological limit to the efficiency of multi-species food webs.

## Supporting Information

S1 Text Relative abundance data for Wabash River fishes from 1974–2008. (XLSX)
